# Comparative Genomics Integrated with Association Analysis Identifies Candidate Effector Genes Corresponding to *Lr20* in Phenotype-Paired *Puccinia triticina* Isolates from Australia

**DOI:** 10.3389/fpls.2017.00148

**Published:** 2017-02-09

**Authors:** Jing Qin Wu, Sharadha Sakthikumar, Chongmei Dong, Peng Zhang, Christina A. Cuomo, Robert F. Park

**Affiliations:** ^1^Faculty of Agriculture and Environment, Plant Breeding Institute, The University of SydneyNarellan, NSW, Australia; ^2^Genome Sequencing and Analysis Program, Broad Institute of Massachusetts Institute of Technology (MIT) and HarvardCambridge, MA, USA

**Keywords:** wheat leaf rust, *Lr20*, effectors, resistance, secreted proteins, genetic association, comparative genetics, avirulence gene

## Abstract

Leaf rust is one of the most common and damaging diseases of wheat, and is caused by an obligate biotrophic basidiomycete, *Puccinia triticina* (*Pt*). In the present study, 20 *Pt* isolates from Australia, comprising 10 phenotype-matched pairs with contrasting pathogenicity for *Lr20*, were analyzed using whole genome sequencing. Compared to the reference genome of the American *Pt* isolate 1-1 BBBD Race 1, an average of 404,690 single nucleotide polymorphisms (SNPs) per isolate was found and the proportion of heterozygous SNPs was above 87% in the majority of the isolates, demonstrating a high level of polymorphism and a high rate of heterozygosity. From the genome-wide SNPs, a phylogenetic tree was inferred, which consisted of a large clade of 15 isolates representing diverse presumed clonal lineages including 14 closely related isolates and the more diverged isolate 670028, and a small clade of five isolates characterized by lower heterozygosity level. Principle component analysis detected three distinct clusters, corresponding exactly to the two major subsets of the small clade and the large clade comprising all 15 isolates without further separation of isolate 670028. While genome-wide association analysis identified 302 genes harboring at least one SNP associated with *Lr20* virulence (*p* < 0.05), a Wilcoxon rank sum test revealed that 36 and 68 genes had significant (*p* < 0.05) and marginally significant (*p* < 0.1) differences in the counts of non-synonymous mutations between *Lr20* avirulent and virulent groups, respectively. Twenty of these genes were predicted to have a signal peptide without a transmembrane segment, and hence identified as candidate effector genes corresponding to *Lr20*. SNP analysis also implicated the potential involvement of epigenetics and small RNA in *Pt* pathogenicity. Future studies are thus warranted to investigate the biological functions of the candidate effectors as well as the gene regulation mechanisms at epigenetic and post-transcription levels. Our study is the first to integrate phenotype-genotype association with effector prediction in *Pt* genomes, an approach that may circumvent some of the technical difficulties in working with obligate rust fungi and accelerate avirulence gene identification.

## Introduction

Wheat leaf rust is caused by *Puccinia triticina* (*Pt*), an obligate biotrophic basidiomycete. Being one of the most common and damaging diseases of wheat, leaf rust can lead to yield losses of more than 15% (Roelfs, 1995). To prevent yield loss, resistance (R) genes have been deployed in wheat varieties, an approach that has proven to be economical and effective. To date, more than 70 leaf rust resistance genes (*Lr*) have been officially designated (McIntosh et al., 1995, 2008), of which *Lr1, Lr3a, Lr10*, and *Lr20* are the most prevalent worldwide (Dakouri et al., 2013). However, many of these resistance genes have been overcome by the evolution of new pathogen pathotypes with matching virulence. The evolution of such pathotypes following the release of varieties containing single *Lr* genes was documented in Australia by Park et al. (1995). Globally, *Pt* populations are highly diverse in virulence phenotype, as exemplified by the detection of a range of different pathotypes annually in the US (Kolmer et al., 2007), Europe (Park et al., 2001; Goyeau et al., 2006), and Australia (Park, 1996). Gaining a better understanding of the genetic basis of phenotypic variations and *Pt*-wheat interactions is crucial for the development of varieties with durable resistance as well as sustained control of wheat leaf rust (Webb and Fellers, 2006).

One of the most widely accepted models of plant-pathogen co-evolution is the gene-for-gene hypothesis, which states that for each gene that conditions resistance in the host there is a corresponding gene that conditions pathogenicity in the parasite (Flor, 1971). In the pathosystem of flax rust *Melampsora lini* (*M. lini*)-flax (*Linum usitatissimum*), Flor originally demonstrated the existence of avirulence (AVR) genes. These avirulence genes produce proteins, also known as effectors, that upon recognition by the host trigger the resistance response. This effector-triggered immunity (ETI) is characterized by accelerated and amplified response normally resulting in a hypersensitive cell death as compared to the pathogen-associated molecular pattern (PAMP)-triggered immunity (PTI; Jones and Dangl, 2006). By mutating to virulence (VIR), the pathogen can evade host recognition and the resistant response is not triggered. To promote virulence and compatibility, fungal effectors target various biological processes important to the plant host, such as the ubiquitin-proteasome system, plant chitin receptor, and jasmonate signaling pathway (De Wit et al., 2009; Lo Presti et al., 2015). In rust pathogens, only a few such effectors have been identified, including: (1) rust transferred protein 1 (RTP1) from bean rust *Uromyces fabae*, which has protease inhibitor activity and is involved in fibril formation (Kemen et al., 2005, 2013; Pretsch et al., 2013); (2) AvrM, AvrL567, AvrP123, and AvrP4 from flax rust *M. lini*, which are all haustorially expressed secreted proteins with AvrP123 containing a Kazal Ser protease inhibitor signature (Dodds et al., 2004; Catanzariti et al., 2006; Upadhyaya et al., 2014b); and (3) PGTAUSPE-10-1 from the wheat stem rust fungus *P. graminis* f. sp. *tritici* (*Pgt*), which triggers cell death in host lines carrying *Sr22* when delivered into wheat cells (Upadhyaya et al., 2014b).

The advent of next generation sequencing technology accompanied by rapidly decreasing cost has dramatically increased the number of available fungal pathogen genome sequences and enabled genome-wide prediction of fungal effectors at a wide-scale and facilitated comparative genomics studies. For instance, the avirulence gene Avr5 of the tomato leaf mold pathogen *Cladosporium fulvum* and AVRFOM2 of the melon wilt fungus *Fusarium oxysporum* f. sp. *melonis* (Fom) were recently identified by comparative genomic approaches (Mesarich et al., 2014; Schmidt et al., 2016). While these effectors as well as those from powdery mildew (Godfrey et al., 2010; Spanu et al., 2010) are all found to share conserved sequences in the N-terminal regions of effector genes, these motifs do not appear to define major classes of effectors in rust fungi (Duplessis et al., 2011; Garnica et al., 2013). Given the lack of conserved motifs, the identification of fungal effectors has been largely based on the broad criteria of protein localization sequences, including presence of signal peptide and absence of transmembrane segment (Sperschneider et al., 2015). Although previous studies have used the small size of protein (< 300 amino acid) as a criterion, this threshold would miss larger effector proteins, such as AvrM (Ravensdale et al., 2011). Despite the difficulties arising from a lack of motif pattern and those arising from the inability to grow obligate biotrophs readily *in vitro*, a recent study attempted to overcome these limitations by detecting associations between 97 secreted protein-single nucleotide polymorphism (SNP) markers and virulence phenotype in a *Puccinia striiformis* f. sp. *tritici* (*Pst*) population (Xia et al., 2016). The successful identification of significantly associated SNP markers for a panel of avirulence genes in *Pst* has demonstrated that association analysis can be useful in identifying candidate avirulence genes. However, this study was limited by the selected number of SNP markers and unbalanced distribution of pathogenicity for specific resistance genes among the panel of isolates used.

With the availability of reference genomes for species within the *Pucciniales*, several resequencing projects have been undertaken, including 15 isolates of *Melampsora larici-populina* from diverse populations collected over 20 years in France (Persoons et al., 2014), 5 isolates of *Pgt* collected over 40 years in Australia (Upadhyaya et al., 2014a), and 4 and 6 isolates of *Pst* resequenced by Cantu et al. (2013) and Zheng et al. (2013), respectively. These studies revealed high genome heterozygosity and variation across the rust pathogens and identified panels of candidate effectors for certain avirulence genes. Although transcriptome profiling of 6 *Pt* races has been used to identify the effective secretomes (Bruce et al., 2014) and resequencing of 120 *Pt* isolates from North America and Europe is underway (Duplessis et al., 2014), no genome resequencing studies of *Pt* have been published so far.

In the present study, we report the genome re-sequencing of 20 Australian *Pt* isolates, comprising 10 pairs of isolates differing in avirulence/virulence only to *Lr20*. The reads of each isolate were mapped against the *Pt* Race 1 reference genome; this high quality draft genome assembly includes a RNA-Seq based annotated gene set as well as the accompanying transcriptome and proteome studies based on the predicted products (Song et al., 2011; Bruce et al., 2014; Rampitsch et al., 2015; Cuomo et al., 2016). For the untranslated region (UTR), a detailed investigation of UTR length in *Pt* has not been carried out, so the selection of 1000 bases upstream and downstream of the coding sequences (CDS) was used to approximate UTRs, which may contain some intergenic sequences. Our analyses not only revealed high genetic variation and heterozygosity rate across the 20 *Pt* genomes, they also predicted a small number of candidate effectors related to the resistance gene *Lr20* using association analysis. Our study is the first to use phenotype-genotype association as a new filter to prioritize candidate effectors of interest at a genome-wide scale through careful design of matched virulence profile, benefiting from nationwide race *Pt* surveys in Australia since 1921. This pilot study suggests a feasible approach to identify and characterize AVR genes, which may greatly accelerate genetic analysis of pathogen populations by circumventing the technical difficulties associated with obligate rust fungi.

## Results

### The alignment of genome sequencing data

**Figure 1:**
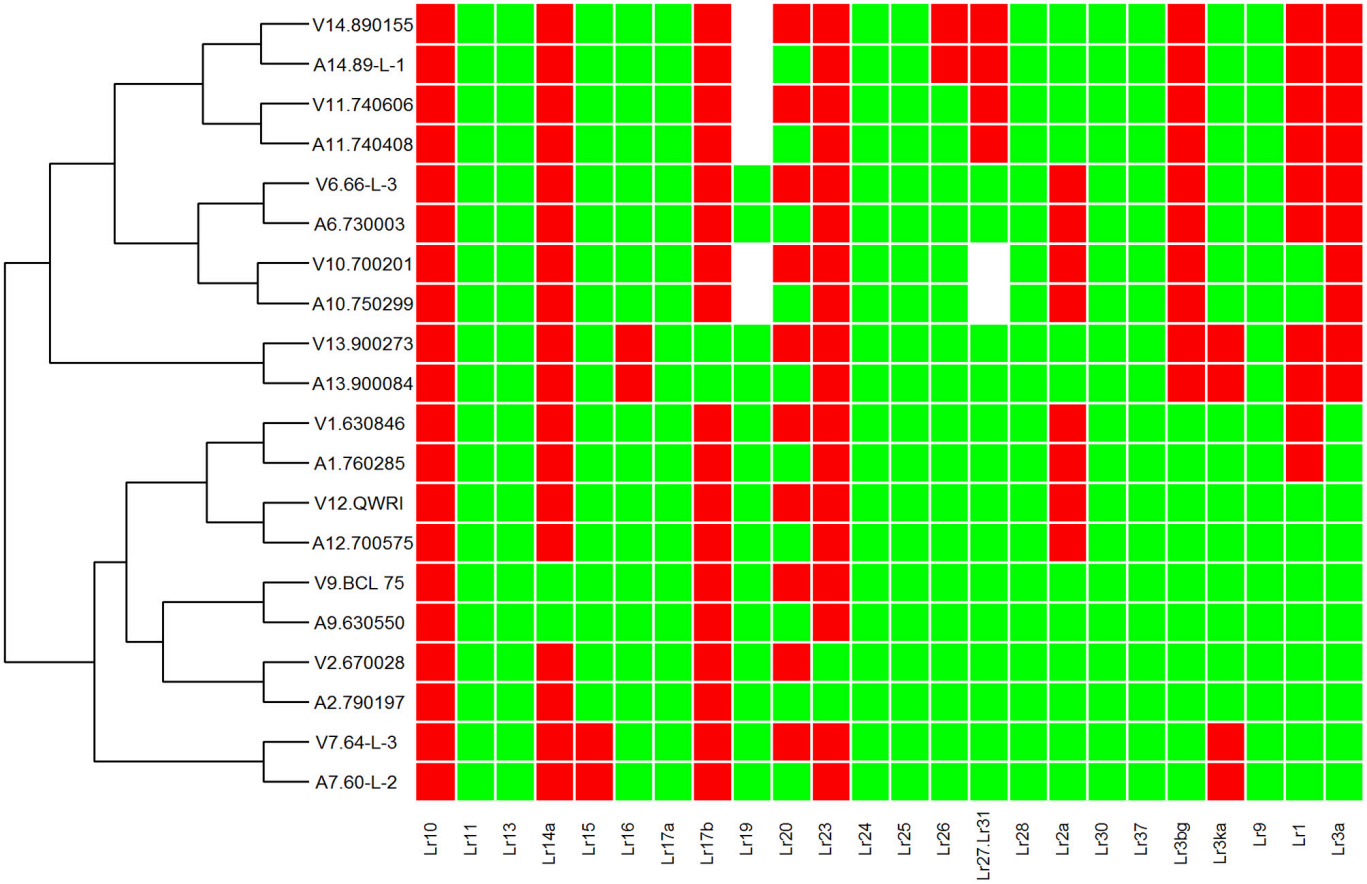
**Heat map and race relationship of 20 ***Pt*** isolates according to their virulence on 24 leaf rust resistance genes**. The susceptible and resistant types are represented by green and red, respectively. Unknown Isolate types for a particular *Lr* gene are represented by white color. “V” and “A” at the beginning of each row label represent the groups of avirulence and virulence to *Lr20*, respectively.

To investigate genetic variation among Australian isolates of *Pt*, whole genome sequence data were generated from 20 DNA samples as 101 base paired-end reads on an Illumina HiSeq2000 platform. Genomic DNA was extracted from urediniospores of the 10 pairs of *Pt* isolates established from single pustules, with each pair comprising two pathotypes differing in pathogenicity only for avirulence/virulence to *Lr20*. For 23 other *Lr* genes, the isolate pairs showed various virulence/avirulence profiles on the cataloged resistance genes present in standard differential genotypes (10, 26, 68, 104, 122, 135, and 162; Table 1) and additional differentials (Figure 1). Overall, 47–88 million reads per sample (Table 2) were obtained and mapped to the draft genome of the American *Pt* isolate 1-1 BBBD Race 1 (Cuomo et al., 2016; version 2; 14,818 supercontigs, 135,343,689 bp; https://www.ncbi.nlm.nih.gov/assembly/GCA_000151525.2). The median aligned read depth was 38.6 fold and the minimum and maximum depth was 27.5 and 49.6-fold, respectively (Table 2). For each isolate, between 74 and 81% of the sequence reads were mapped to the race 1 genome, which covered between 97.3 and 98.5% of the reference genome bases. Our subsequent analysis focused on the alignment data against this reference genome, as it not only covered the major part of our data, but this sequence also had annotations with transcriptome and proteome studies that facilitated further biological interpretations (Song et al., 2011; Bruce et al., 2014; Rampitsch et al., 2015).

**Table 1:** **Ten pairs of ***Puccinia triticina*** isolates and the associated avirulent/virulent profiles**.

**Isolate**	**Pair**	**Pathogenicity for Lr20**	**Pathotype**	**Virulent**	**Avirulent**	**Collection year**	**Collection location**	**Region**
760285	1	Avirulence	10-2,3	Lr1, Lr2a, Lr10, Lr14a, Lr17b, Lr23,	Lr3a, Lr3bg, Lr3ka, Lr9, Lr11, Lr13, Lr15, Lr16, Lr17a, Lr19, Lr20, Lr24, Lr25, Lr26, Lr27+Lr31, Lr28, Lr30, Lr37	1976	Coorangy, QLD	1
630846	1	Virulence	10-1,2,3	Lr1, Lr2a, Lr10, Lr14a, Lr17b, Lr20, Lr23,	Lr3a, Lr3bg, Lr3ka, Lr9, Lr11, Lr13, Lr15, Lr16, Lr17a, Lr19, Lr24, Lr25, Lr26, Lr27+Lr31, Lr28, Lr30, Lr37	1980	Gurley (fairall)	1
790197	2	Avirulence	26-3	Lr10, Lr14a, Lr17b	Lr1, Lr2a, Lr3a, Lr3bg, Lr3ka, Lr9, Lr11, Lr13, Lr15, Lr16, Lr17a, Lr19, Lr20, Lr23, Lr24, Lr25, Lr26, Lr27+Lr31, Lr28, Lr30, Lr37	1979	Bookpurnong, SA	3
670028	2	Virulence	26-1,3	Lr10, Lr14a, Lr17b, Lr20	Lr1, Lr2a, Lr3a, Lr3bg, Lr3ka, Lr9, Lr11, Lr13, Lr15, Lr16, Lr17a, Lr19, Lr23, Lr24, Lr25, Lr26, Lr27+Lr31, Lr28, Lr30, Lr37	1980	Takaka, NZ	9
730003	6	Avirulence	122-2,3	Lr1, Lr2a, Lr3a, Lr3bg, Lr10, Lr14a, Lr17b, Lr23	Lr3ka, Lr9, Lr11, Lr13, Lr15, Lr16, Lr17a, Lr19, Lr20, Lr24, Lr25, Lr26, Lr27+Lr31, Lr28, Lr30, Lr37	1974	University of New England,NSW	1
66-L-3	6	Virulence	122-1,2,3	Lr1, Lr2a, Lr3a, Lr3bg, Lr10, Lr14a, Lr17b, Lr20, Lr23	Lr3ka, Lr9, Lr11, Lr13, Lr15, Lr16, Lr17a, Lr19, Lr24, Lr25, Lr26, Lr27+Lr31, Lr28, Lr30, Lr37	1974	Castle Hill Glasshouse	2
60-L-2	7	Avirulence	135-2,3,4,5	Lr3ka, Lr10, Lr14a, Lr15, Lr17b, Lr23	Lr1, Lr2a, Lr3a, Lr3bg, Lr9, Lr11, Lr13, Lr16, Lr17a, Lr19, Lr20, Lr24, Lr25, Lr26, Lr27+Lr31, Lr28, Lr30, Lr37	1978	Castle Hill Glasshouse	2
64-L-3	7	Virulence	135-1,2,3,4,5	Lr3ka, Lr10, Lr14a, Lr15, Lr17b, Lr20, Lr23	Lr1, Lr2a, Lr3a, Lr3bg, Lr9, Lr11, Lr13, Lr16, Lr17a, Lr19, Lr24, Lr25, Lr26, Lr27+Lr31, Lr28, Lr30, Lr37	1976	Castle Hill Glasshouse	2
630550	9	Avirulence	135-2	Lr10, Lr17b, Lr23	Lr1, Lr2a, Lr3a, Lr3bg, Lr3ka, Lr9, Lr11, Lr13, Lr14a, Lr15, Lr16, Lr17a, Lr19, Lr20, Lr24, Lr25, Lr26, Lr27+Lr31, Lr28, Lr30, Lr37	1974	Ravensworth, NSW	2
BCL 75	9	Virulence	135-1,2	Lr10, Lr17b, Lr20, Lr23	Lr1, Lr2a, Lr3a, Lr3bg, Lr3ka, Lr9, Lr11, Lr13, Lr14a, Lr15, Lr16, Lr17a, Lr19, Lr24, Lr25, Lr26, Lr27+Lr31, Lr28, Lr30, Lr37	1974	University of Sydney	2
750299	10	Avirulence	162-2,3,6	Lr2a, Lr3a, Lr3bg, Lr10, Lr14a, Lr17b, Lr23	Lr1, Lr3ka, Lr9, Lr11, Lr13, Lr15, Lr16, Lr17a, Lr20, Lr24, Lr25, Lr26, Lr27+Lr41, Lr28, Lr30, Lr37	1976	Wandoan, QLD	1
700201	10	Virulence	162-1,2,3,6	Lr2a, Lr3a, Lr3bg, Lr10, Lr14a, Lr17b, Lr20, Lr23	Lr1, Lr3ka, Lr9, Lr11, Lr13, Lr15, Lr16, Lr17a, Lr24, Lr25, Lr26, Lr27+Lr41, Lr28, Lr30, Lr37	1974	QWRI, QLD	1
740408	11	Avirulence	104-2,3,6	Lr1, Lr3a, Lr3bg, Lr10, Lr14a, Lr17b, Lr23, Lr27+Lr31	Lr2a, Lr3ka, Lr9, Lr11, Lr13, Lr15, Lr16, Lr17a, Lr20, Lr24, Lr25, Lr26, Lr28, Lr30, Lr37	1975	Biloela, QLD	1
740606	11	Virulence	104-1,2,3,6	Lr1, Lr3a, Lr3bg, Lr10, Lr14a, Lr17b, Lr20, Lr23, Lr27+Lr31	Lr2a, Lr3ka, Lr9, Lr11, Lr13, Lr15, Lr16, Lr17a, Lr24, Lr25, Lr26, Lr28, Lr30, Lr37	1975	Narrabri, NSW	1
700575	12	Avirulence	68-2,3	Lr2a, Lr10, Lr14a, Lr17b, Lr23	Lr1, Lr3a, Lr3bg, Lr3ka, Lr9, Lr11, Lr13, Lr15, Lr16, Lr17a, Lr19, Lr20, Lr24, Lr25, Lr26, Lr27+Lr31, Lr28, Lr30, Lr37	1974	Wellington, NZ	9
QWRI	12	Virulence	68-1,2,3,4	Lr2a, Lr10, Lr14a, Lr17b, Lr20, Lr23	Lr1, Lr3a, Lr3bg, Lr3ka, Lr9, Lr11, Lr13, Lr15, Lr16, Lr17a, Lr19, Lr24, Lr25, Lr26, Lr27+Lr31, Lr28, Lr30, Lr37	1990	QWRI, QLD	1
900084	13	Avirulence	104-2,3,5,(6),(7),11	Lr1, Lr3a, Lr3bg, Lr3ka, Lr10, Lr14a, Lr16, Lr23	Lr2a, Lr9, Lr11, Lr13, Lr15, Lr17a, Lr17b, Lr19, Lr20, Lr24, Lr25, Lr26, Lr27+Lr31, Lr28, Lr30, Lr37	1991	PBI Cobbitty, Lansdowne	2
900273	13	Virulence	104-1,2,3,5,(6),(7),11	Lr1, Lr3a, Lr3bg, Lr3ka, Lr10, Lr14a, Lr16, Lr20, Lr23	Lr2a, Lr9, Lr11, Lr13, Lr15, Lr17a, Lr17b, Lr19, Lr24, Lr25, Lr26, Lr27+Lr31, Lr28, Lr30, Lr37	1991	Mt Ridley	2
89-L-1	14	Avirulence	104-2,3,6,(7),9	Lr1, Lr3a, Lr3bg, Lr10, Lr14a, Lr17b, Lr23, Lr26, Lr27+Lr31	Lr2a, Lr3ka, Lr9, Lr11, Lr13, Lr15, Lr16, Lr17a, Lr20, Lr24, Lr25, Lr28, Lr30, Lr37	1989	Castle Hill Glasshouse	2
890155	14	Virulence	104-1,2,3,6,(7),(9)	Lr1, Lr3a, Lr3bg, Lr10, Lr14a, Lr17b, Lr20, Lr23, Lr26, Lr27+Lr31	Lr2a, Lr3ka, Lr9, Lr11, Lr13, Lr15, Lr16, Lr17a, Lr24, Lr25, Lr28, Lr30, Lr37	1990	Tamworth	1

**Table 2:** **General mapping information for the 20 ***Puccinia triticina*** isolates**.

**Isolate**	**Pair**	**Total reads**	**Mapped reads**	**% Mapped reads**	**Average depth of cover**	**% of reference covered by the reads**
760285	1	51,764,276	40,827,968	78.9	28.9	97.6
630846	1	70,048,288	55,559,224	79.3	38.8	97.6
790197	2	71,405,410	56,812,142	79.6	39.6	97.3
670028	2	71,622,608	57,466,213	80.2	40	97.7
730003	6	74,683,730	55,381,229	74.2	38.7	97.7
66-L-3	6	68,912,142	55,179,318	80.1	38.6	97.5
60-L-2	7	47,791,600	38,719,913	81.0	27.5	97.5
64-L-3	7	88,145,358	71,086,601	80.6	49.6	97.6
630550	9	67,044,878	53,907,198	80.4	37.6	97.5
BCL 75	9	61,007,614	49,381,730	80.9	34.6	97.3
750299	10	72,470,524	57,394,609	79.2	40.1	97.9
700201	10	69,898,526	55,865,395	79.9	38.9	98.5
740408	11	74,849,258	60,626,983	81.0	42.3	97.9
740606	11	74,179,490	59,062,056	79.6	41.1	97.8
700575	12	58,917,520	47,593,247	80.8	33.5	97.9
QWRI	12	62,858,660	47,787,010	76.0	33.3	97.8
900084	13	53,552,614	43,178,267	80.6	30.3	97.6
900273	13	64,944,794	49,761,366	76.6	34.8	97.8
89-L-1	14	69,516,584	55,126,898	79.3	38.4	97.9
890155	14	68,046,788	51,725,917	76.0	36.0	98.5

### Genome-wide genotyping of the 20 *Pt* isolates

To obtain a detailed view of the sequence variation among the isolates, SNPs were identified for the 20 *Pt* isolates by mapping the sequence reads of each isolate independently against the race 1 reference genome. An average of 404,690 SNPs per isolate was found, which covered 0.30% of the reference genome. Across all isolates, the number of SNPs identified was around 400,000 except the two isolates in pair 7, which had < 350,000 SNPs (Table 3). When the 20 genomes were considered together, 306,474 SNP sites including both homozygous and heterozygous states were identified in all of the isolates, consisting of 44.2% of the total 693,101 SNP positions concatenated from the 20 isolates. These SNPs, along with the two allele genotype calls, are shown in Table 1 of Additional File 1. Out of the 306,474 SNP sites, 119,962 sites had a homozygous reference matching genotype in at least one isolate, whereas the remaining 186,512 sites did not show any homozygous genotype of reference alleles in any isolate. While the heterozygosity rate of SNP positions in a majority of the isolates was above 87%, it was lower in pair 7, pair 13, and isolate 89-L-1 in pair 14, ranging from 72.5 to 81.0% (Table 3). Polymorphic sites within CDS and intergenic regions represented ~11 and 83% of total SNPs, respectively, and these percentages were consistent across the isolates (Table 3). For SNPs within a gene, the percentages of non-synonymous and synonymous SNPs also appeared homogeneous across the isolates, ranging from 60.5 to 61.2 and 36.7 to 37.4%, respectively. The SNP frequency was higher in introns (4.2 SNPs/kb) than in exons (2.0 SNPs/kb) and the ratio of SNPs in coding vs. intronic regions had little variation (~3.5) across the pathotypes (Table 3). SNPs were also inspected in the 1 kb upstream (5′ UTR) and downstream regions (3′ UTR) of CDS, where they may have a regulatory role at the transcription and post-transcription level, respectively. The selection of 1 kb upstream and downstream of the CDS as UTRs, due to the lack of the detailed information of UTR in *Pt*, may include some intergenic sequences. Of the total SNPs identified in each pathotype, about 2.1% were located in the UTR region and the ratio of SNPs in 3′ vs. 5′ UTR was about 1.8 across all pathotypes (Table 3).

**Table 3:** **Total number and type of SNPs in the 20 ***Pt*** isolates**.

**Isolate**	**SNPs**	**Ref.homo**	**Data.missing**	**Intergenic**	**Intronic**	**p5UTR**	**p3UTR**	**Coding**	**SYN**	**NSY**	**Read-Through**	**Nonsense**	**Homozygous**	**Heterozygous**	**% Intergenic**	**% coding**	**coding versus intronic**	**% SYN**	**% NSY**	**% homozygous**	**% heterozygous**	**3UTR vs 5UTR**
760285	398,470	105,557	189,074	332,036	12,804	3032	5566	45,032	16,756	27,346	177	753	50,091	348,379	83.3	11.3	3.52	37.2	60.7	12.6	87.4	1.84
630846	406,525	142,134	144,442	339,081	13,032	3046	5718	45,648	16,966	27,764	186	732	51,387	355,138	83.4	11.2	3.50	37.2	60.8	12.6	87.4	1.88
790197	408,240	143,658	141,203	340,482	13,090	3088	5692	45,888	17,034	27,927	179	748	51,164	357,076	83.4	11.2	3.51	37.1	60.9	12.5	87.5	1.84
670028	407,573	145,214	140,314	340,042	12,998	3094	5646	45,793	17,009	27,842	180	762	50,900	356,673	83.4	11.2	3.52	37.1	60.8	12.5	87.5	1.82
730003	407,980	142,704	142,417	340,387	13,005	3085	5650	45,853	17,026	27,876	180	771	51,068	356,912	83.4	11.2	3.53	37.1	60.8	12.5	87.5	1.83
66-L-3	406,826	141,290	144,985	339,294	13,070	3065	5658	45,739	17,000	27,801	176	762	51,046	355,780	83.4	11.2	3.50	37.2	60.8	12.5	87.5	1.85
60-L-2	329,330	138,842	224,929	275,507	10,245	2401	4521	36,656	13,457	22,426	161	612	71,613	257,717	83.7	11.1	3.58	36.7	61.2	21.7	78.3	1.88
64-L-3	341,857	231,639	119,605	286,364	10,622	2485	4680	37,706	13,820	23,087	159	640	93,995	247,862	83.8	11.0	3.55	36.7	61.2	27.5	72.5	1.88
630550	405,461	140,326	147,314	338,131	13,039	3105	5679	45,507	16,898	27,691	179	739	51,021	354,440	83.4	11.2	3.49	37.1	60.8	12.6	87.4	1.83
BCL 75	402,732	131,151	159,218	335,854	12,884	3051	5644	45,299	16,768	27,590	184	757	50,794	351,938	83.4	11.2	3.52	37.0	60.9	12.6	87.4	1.85
750299	409,340	145,799	137,962	341,488	13,118	3059	5709	45,966	17,021	27,990	181	774	51,282	358,058	83.4	11.2	3.50	37.0	60.9	12.5	87.5	1.87
700201	409,220	142,703	141,178	341,472	13,034	3109	5678	45,927	17,059	27,941	179	748	51,747	357,473	83.4	11.2	3.52	37.1	60.8	12.6	87.4	1.83
740408	410,194	150,429	132,478	342,294	13,113	3094	5678	46,015	17,030	28,060	181	744	51,371	358,823	83.4	11.2	3.51	37.0	61.0	12.5	87.5	1.84
740606	409,683	147,405	136,013	341,821	13,075	3086	5705	45,996	17,073	27,983	184	756	51,162	358,521	83.4	11.2	3.52	37.1	60.8	12.5	87.5	1.85
700575	404,032	125,345	163,724	336,920	12,925	3064	5632	45,491	16,911	27,631	178	771	50,727	353,305	83.4	11.3	3.52	37.2	60.7	12.6	87.4	1.84
QWRI	402,731	125,706	164,664	335,748	12,885	3068	5627	45,403	16,861	27,623	177	742	50,179	352,552	83.4	11.3	3.52	37.1	60.8	12.5	87.5	1.83
900084	435,758	95,663	161,680	364,218	13,846	3274	5929	48,491	18,119	29,320	197	855	82,591	353,167	83.6	11.1	3.50	37.4	60.5	19.0	81.0	1.81
900273	443,430	107,865	141,806	370,693	14,034	3288	6048	49,367	18,365	29,943	192	867	85,005	358,425	83.6	11.1	3.52	37.2	60.7	19.2	80.8	1.84
89-L-1	446,048	117,590	129,463	372,899	14,131	3328	6057	49,633	18,478	30,076	195	884	85,572	360,476	83.6	11.1	3.51	37.2	60.6	19.2	80.8	1.82
890155	408,368	133,523	151,210	340,839	13,002	3129	5610	45,788	17,002	27,825	182	779	51,566	356,802	83.5	11.2	3.52	37.1	60.8	12.6	87.4	1.79

*Ref.homo, homozygous reference alleles for the genotype calling*.

*Data.missing, missing data at the SNP sites concatenated from all of the 20 isolates*.

*For each isolate, number of SNPs, Ref.homo, and Data.missing added up to 693,101 SNPs concatenated from all of the isolates*.

*UTR, untranslated region; SYN, Synonymous SNPs; NSY, Non-synonymous SNPs*.

SNPs, total SNP identified in an isolate, which included intergenic, intronic, SNPs in UTRs and in coding regions;

*SNPs in coding regions were further classified as SYN, NSY, read-through, and nonsense SNPs*.

*% Intergenic, Intergenic SNPs/total SNPs identified per isolate; % coding, Coding SNPs/total SNPs identified per isolate*.

*% NSY, percentage of Non-synonymous SNPs/Coding SNPs; % SYN, percentage of Synonymous SNPs/Coding SNPs*.

### Phylogenetic tree based on whole genome SNPs

**Figure 2:**
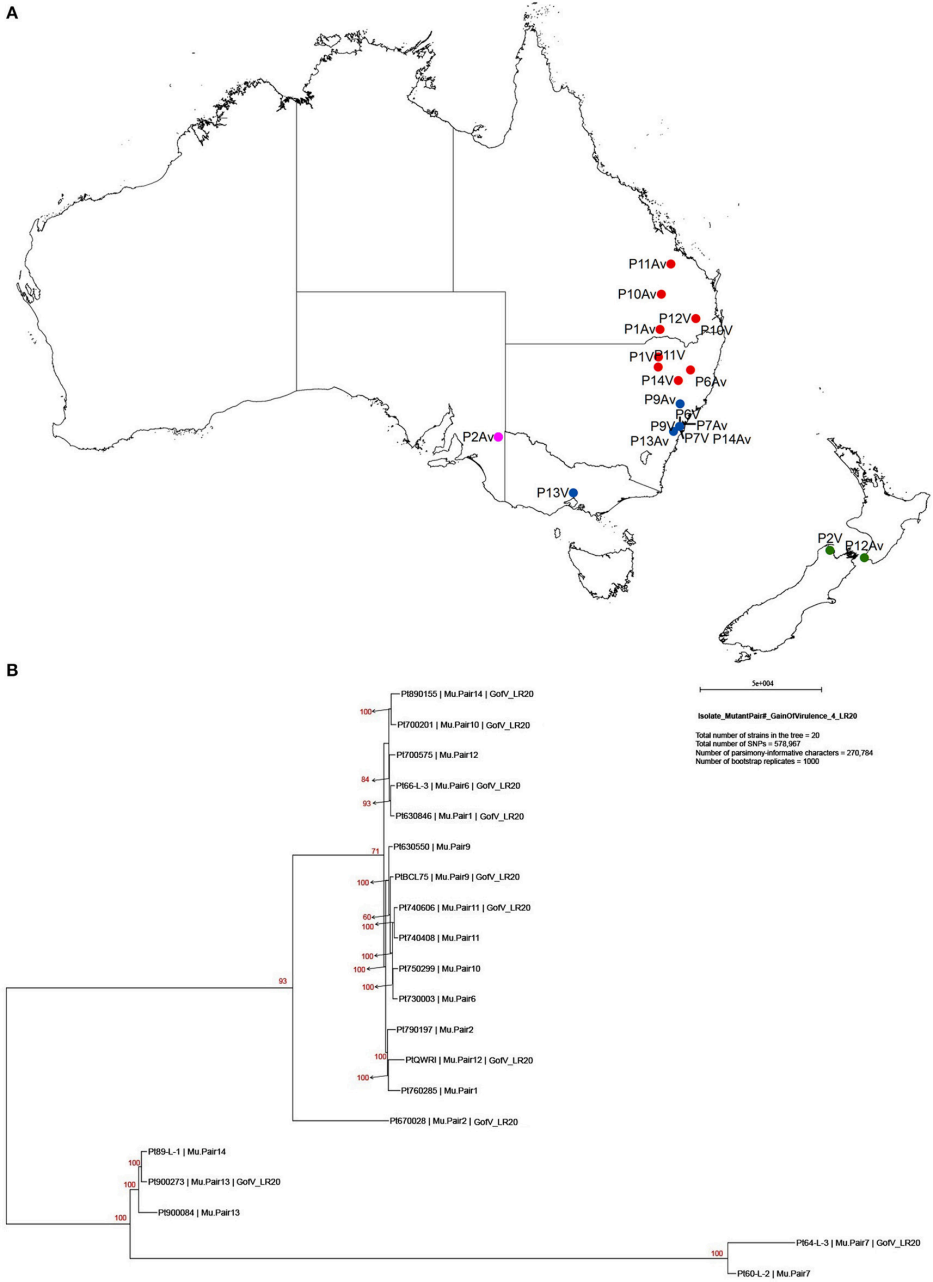
Demographic map showing collection sites and phylogenic tree of 20 ***Pt*** isolates constructed with 578,967 SNPs by PAUP^*^. (A) Demographic map showing location and region of sample collection sites. Red, blue, pink, and green dots represent region 1, 2, 3, and 9, respectively. P represents for the pair, Av for avirulent on *Lr20*, and V for virulent on *Lr20*. For example, P11Av represents for the avirulent isolate of pair 11. **(B)** Maximum Parsimony and “hetequal” substitution model were used and 1000 bootstrap replicates were applied for tree building. Each isolate was labelled in order of isolate number, mutant pair number, and virulence to *Lr20*. Gain of virulence of *Lr20* was abbreviated as “GofV_LR20.”

Previous studies on comparisons of pathogenicity suggest that these isolates belong to four of five clonal lineages of *Pt* detected in Australia between 1921 and 1984, viz. Lineage 1 (mutant pairs 7, 9, 11, and 14); Lineage 2 (mutant pair 2); Lineage 3 (mutant pairs 1, 6, 10, 12); and Lineage 5 (mutant pair 13; Park et al., 1995; Park RF unpublished; Luig NH unpublished). Most isolates were collected from sites in Australia with two from New Zealand, as shown in a demographic map (Figure 2A). Phylogenetic analysis revealed that while a majority of the isolates including those from Lineages 1, 2, and 3 formed one large clade with isolate 670028 more divergent from the others, both pair 13 isolates (900273 and 900084) and one pair 14 isolate (89-L-1) along with both pair 7 isolates (64-L-3 and 60-L-2) formed a separate clade well separated from all the remaining isolates (Figure 2B). Principle component analysis (PCA) showed 3 distinct clusters (Figure 1 in Additional File 1), corresponding to the two major subsets of the small clade and the large clade, respectively. Within the large cluster of PCA, consistent with the large clade in the phylogenetic tree, the same 15 isolates were closely related to each other without further separation of isolate 670028. While all these results were largely consistent with the genetic relationships imputed from previous studies based on pathogenicity for Lineages 3 and 5, the separation of mutant pair 7 from 9 and of the two isolates within pair 14 across the two major clades indicated that the genetic structure of *Pt* populations in Australia between 1921 and 1984 is somewhat more complex than previously thought. The results did however clearly confirm the very close genetic relationship between eight of the 10 putative mutant pairs (1, 6, 7, 9, 10, 11, 12, and 13; Figure 2B). The wide separation of isolates within mutant pairs 2 and 14 was unexpected, and the reasons for this remain unknown. Although differing regions of collection (Figure 2A) could be a factor, it is likely not the most important one because some other isolates from different regions were closely related (e.g., the sister group consisting of 66-L-3 (region 2) and 630846 (region 1; Table 1; Figure 2B).

### The association between SNP and *Lr20* avirulence/virulence

**Figure 3:**
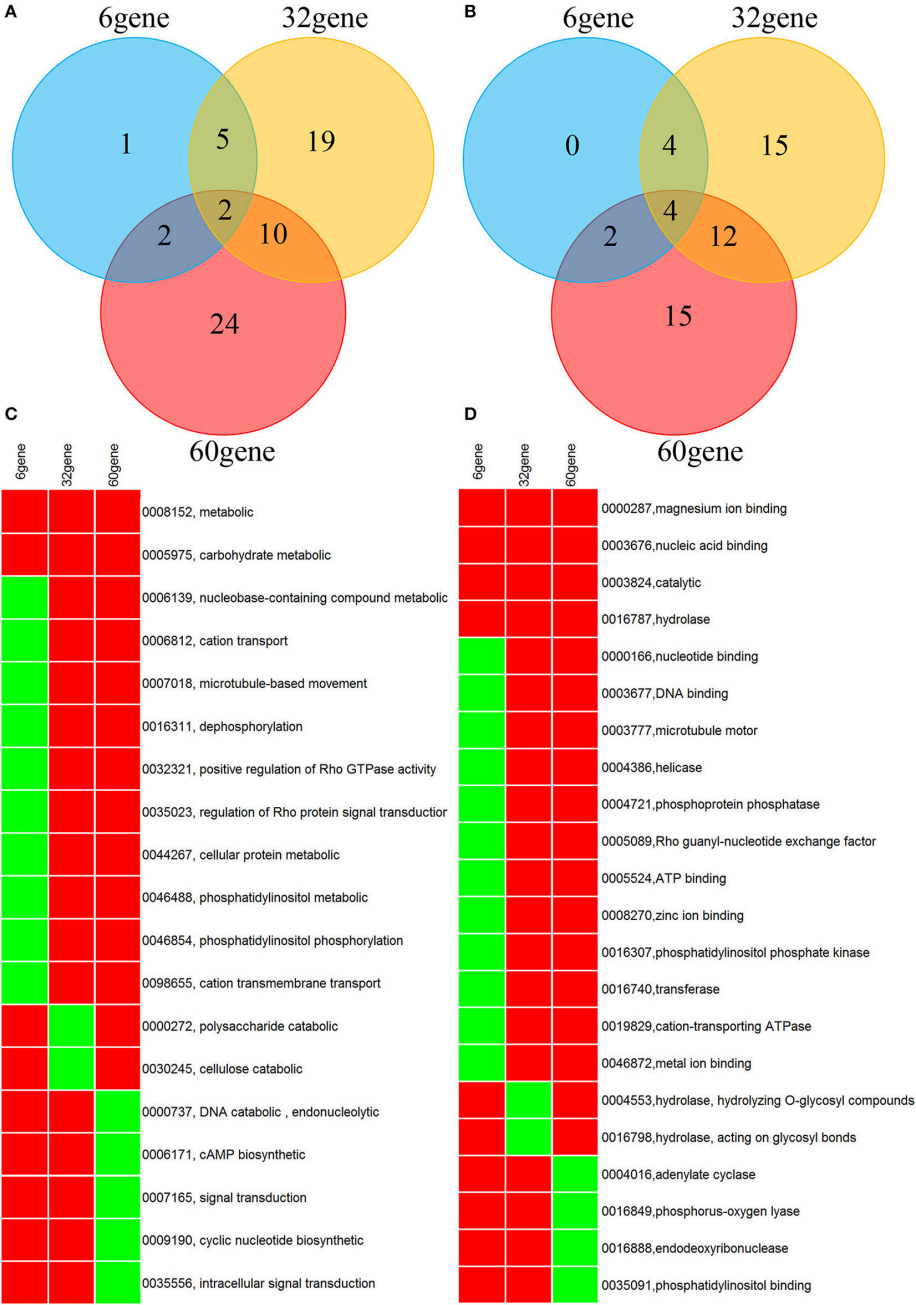
**Venn diagram and heatmap showing GO annotation of the genes derived from SNP association (32 gene), Wilcoxon rank sum test (6 gene), and counts of NSY SNP > 10 (60 gene). (A,B)** Venn diagram for GO categories of biological process and molecular function, respectively. **(C,D)** Heatmap for GO categories of biological process and molecular function, respectively. Only GO terms present in at least 2 of the 3 gene lists are shown. Rows represent GO terms and columns represent genes derived from the aforementioned 3 methods. Red indicates the presence of the GO term in the corresponding gene list, and green indicates absence. The labeling of each row represents both GO ID and its description.

A genome-wide association analysis was attempted using a permutation test implemented in plink to compare the allele frequencies between the *Lr20-*virulent and -avirulent groups. Although our sample size is small, the association findings could be taken as an indicator of potential genetic determinants underpinning pathogenicity, i.e., serving as a new filter to prioritize candidate effectors of interest. First, using genotype calls of both alleles including homozygous and heterozygous states, we identified 3650 SNPs associated with the *Lr20* virulence phenotype (*p* < 0.05), of which 354 SNPs were within an exon and 302 genes were found harboring at least one SNP associated with the *Lr20* virulence (Table 1 in Additional File 2). Of this set, 32 genes (10.6%) could be annotated with potential structural or enzymatic functions, some of which may be relevant to rust pathogenicity, such as signal peptide processing, Golgi organization, and phosphatidylinositol phosphate kinase activity (Table 2 in Additional File 2; Figure 3). Next, we examined whether any of these 302 genes could be potential effectors based on the criteria of the presence of a signal peptide and the absence of a transmembrane segment. A candidate with a PFAM domain of transposable element (gag-polypeptide of long terminal repeats copia-type) was further removed. Eighteen genes met the criteria (Table 4) and both homozygous and heterozygous states were observed for these associated SNP sites. For instance, for the SNP site in PTTG_29866 (5240 bp in supercontig 1058), 3 virulent isolates (740606, 890155, and QWRI) shared the heterozygous genotype of AG that was absent in avirulent isolates, whereas for the SNP site in PTTG_08794 (503523 bp in supercontig 70), 4 virulent isolates (670028, BCL-75, 740606, and QWRI) shared the homozygous genotype of TT that was absent in avirulent isolates. Both of these changes result in amino acid replacement: the former SNP (reference allele A and variant allele G) resulted in an amino acid (aa) change from threonine (codon Act) to alanine (codon Gct), the latter SNP (reference allele T and variant allele C) led to an aa change from aspartic acid (codon gAc) to glycine (codon gGc). Of these 18 candidates, two could be functionally annotated by GO (PTTG_01476 involved in peptidase activity and PTTG_03866 involved in carbohydrate metabolic process) and eight were previously predicted as effectors by a transcriptome study on six races of *Pt* (Table 4; Bruce et al., 2014). Among these eight genes, PTTG_06324 was also supported by proteomic studies (Song et al., 2011; Rampitsch et al., 2015). As candidate effectors are also expected to be specific to individual species or strains, we examined the conservation of these genes in related fungi; 11 of the 18 candidates were conserved in other Basidiomycete genomes, 4 were *Pt* specific, and 3 were *Puccinia* specific (Table 4; Li et al., 2003; Cuomo et al., 2016).

**Table 4:** **Effector candidates with at least one SNP associated with ***Lr20*** virulence profile or differential counts of non-synonymous SNPs between groups**.

**Name**	**Chr**	**Start**	**Stop**	**Gene size**	SignalP D score^*^	Conservation^*^	**PFAM domains**	**Association type**
**PTTG_01476**	1	294157	298485	521	0.689	Basidiomycete orthologs^*^	PF00026.18 Eukaryotic aspartyl protease	SNP association
PTTG_25257	2	1370524	1371097	125	0.631	Pt specific, unique	–	SNP association and differential counts of NSY SNPs
**PTTG_25496**	5	166645	167710	180	0.667	Pt specific, unique	–	differential counts of NSY SNPs
PTTG_11639	6	869389	870867	226	0.752	Pt specific, unique	–	SNP association
PTTG_06625	18	464196	465868	504	0.847	Puccinia specific	–	SNP association
**PTTG_00930**	19	117505	119219	196	0.815	Basidiomycete orthologs	PF10342.4 Ser-Thr-rich GPI-anchored membrane family	SNP association
**PTTG_00931**	19	119666	121280	209	0.743	Basidiomycete orthologs	PF10342.4 Ser-Thr-rich GPI-anchored membrane family	SNP association
**PTTG_26540**	25	646221	647843	520	0.774	Basidiomycete orthologs	PF12929.2 Stretch-activated Ca2+-permeable channel component	SNP association
**PTTG_03866**	32	424755	427481	499	0.852	Basidiomycete orthologs	–	SNP association
**PTTG_06324**	48	110274	111930	296	0.711	Basidiomycete orthologs	–	SNP association
**PTTG_06325**	48	98567	99584	280	0.862	Basidiomycete orthologs	–	differential counts of NSY SNPs
PTTG_03715	68	410717	413942	453	0.899	Basidiomycete orthologs	PF02469.17 Fasciclin domain	SNP association
PTTG_08794	70	503111	504331	134	0.915	Puccinia specific	–	SNP association
PTTG_29130	205	39564	40432	278	0.462	Puccinia specific	–	SNP association
PTTG_29551	567	1165	1583	114	0.886	Pt specific, paralog	–	SNP association
**PTTG_09239**	567	5976	7734	190	0.901	Basidiomycete orthologs	PF02221.10 ML domain	SNP association
**PTTG_29866**	1058	3601	6690	805	0.806	Basidiomycete orthologs	–	SNP association
PTTG_03824	2956	59	1354	401	0.657	Pt specific, unique	–	SNP association
PTTG_30373	3202	106	1862	460	0.467	Basidiomycete orthologs	–	SNP association
PTTG_30778	6810	178	872	212	0.523	Basidiomycete orthologs	–	SNP association

*Data are sorted by chromosome coordinates; Chr: chromosome*.

*Genes previously predicted by transcriptome and/or proteome studies are highlighted in bold*.

* *SignalP D scores >0.45 were considered positive predictions*.

* *Conservation assessed for P. graminis f. sp. Tritici, P. striiformis, and 8 other Basidiomycete genomes (Cuomo et al., 2016)*.

* *Basidiomycete orthologs: putative basidiomycete orthologs which were identified by the previous study using software OrthoMCL based on sequence homology only (Cuomo et al., 2016)*.

### Large proportion of genes harbouring non-synonymous SNPs

Overall, 12,669 genes out of the total 14,880 genes (85.3%) had at least one SNP; 2402 and 63 genes accumulated more than 10 and 50 SNPs across the 20 genomes, respectively (Table 1 in Additional File 3). As nonsynonymous (NSY) changes may have more direct functional implications in *Pt* pathogenicity, the dispersion of the NSY changes was further inspected, revealing that 10,044 genes (67.5% of the total) contained at least one NSY mutation and a total of 638 genes from at least one of the 20 isolates had more than 10 NSY SNPs (Table 2 in Additional File 3). When taking gene length into consideration, 645 genes had more than 10 NSY SNPs/kb (Table 3 in Additional File 3). To get a comprehensive understanding of the functional implications of these top ranked genes, the genes with more than 10 NSY SNPs from both raw and normalized counts were further combined. This approach yielded 1027 genes in total, of which 60 could be functionally annotated (5.8%; Table 4 in Additional File 3; Figure 3).

### Genes correlated with *Lr20* avirulence/virulence by differential counts of NSY SNPs

**Figure 4:**
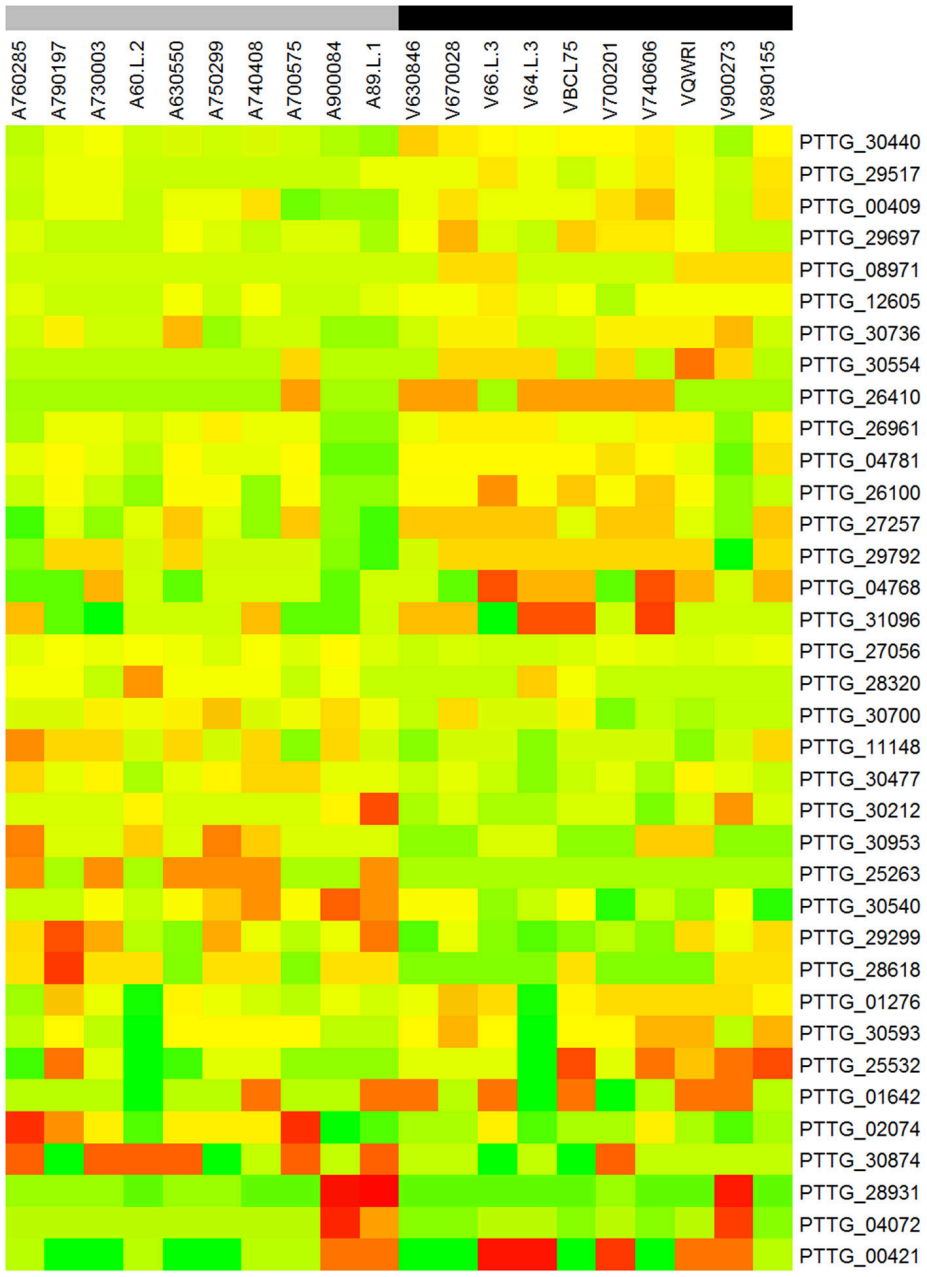
**Heatmap of the 36 genes with differential NSY SNP counts (***p*** < 0.05) between avirulent and virulent groups**. Each row represents a gene and each column represents a sample. For each gene, the various rates of NSY SNP across the 20 isolates were indicated by green, yellow, and red colors corresponding to values ranging from low to high. “A” and “V” at the beginning of each column label represent the groups of avirulence and virulence to *Lr20*, respectively. The gray color of the bar indicates avirulent group and black virulent group.

To identify genes displaying differential patterns of NSY SNPs correlated with *Lr20* avirulence/virulence profile, we compared the total number of NSY mutations including both homozygous and heterozygous genotypes between the AVR and VIR groups. A Wilcoxon rank sum test (a non-parametric test for matched samples) revealed that 36 and 68 genes had significant (*p* < 0.05) and marginally significant (*p* < 0.1; Additional File 4) differences in the counts of NSY mutations between groups, respectively. The heatmap of the 36 genes with differential NSY SNP counts (*p* < 0.05) between avirulent and virulent groups is shown in Figure 4, where for each gene, the various rates of NSY SNP across the 20 isolates are indicated by green, yellow, and red colors corresponding to values ranging from low to high. While 59 genes had a greater number of NSY mutations in the VIR vs. the AVR group, 45 genes had significantly fewer NSY SNPs. Six of the 104 genes (5.8%) could be annotated with potential structural or enzymatic functions (Table 5), and 3 genes (PTTG_25257, PTTG_25496, and PTTG_06325) were predicted as candidate effectors (Table 4). Taken together, the aforementioned list of the candidate effectors was expanded to 20 genes in total with PTTG_25257 present in both predictions (Table 4). PTTG_25496 and PTTG_06325 were also predicted as potential effectors in a previous transcriptome study (Bruce et al., 2014) and two NSY mutations were detected for each of them. While both homozygous and heterozygous genotypes have been observed for each SNP site, it was noted that at the SNP site in PTTG_25496 (166,979 bp in supercontig 5), 7 virulent isolates shared the homozygous genotype of CC, whereas 6 avirulent isolates shared the heterozygous genotype of CT. This SNP (reference allele C and variant allele T) resulted in the aa change from arginine (codon cGa) to glutamine (codon cAa).

**Table 5:** **GO annotation available for the 6 genes out of the 104 genes showing differential NSY SNP counts between ***Lr20*** avirulent and virulent isolates**.

**Gene ID**	**Protein name**	**GO ID**	**GO name**	**GO namespace**
PTTG_01276	Glucanase (EC 3.2.1.-)	GO:0030245	Cellulose catabolic process	BP
PTTG_01276	Glucanase (EC 3.2.1.-)	GO:0000272	Polysaccharide catabolic process	BP
PTTG_01642	Uncharacterized protein	GO:0003676	Nucleic acid binding	MF
PTTG_02074	Uncharacterized protein	GO:0000737	DNA catabolic process, endonucleolytic	BP
PTTG_02074	Uncharacterized protein	GO:0016888	Endodeoxyribonuclease activity, producing 5'-phosphomonoesters	MF
PTTG_03037	Uncharacterized protein	GO:0006171	cAMP biosynthetic process	BP
PTTG_03037	Uncharacterized protein	GO:0009190	Cyclic nucleotide biosynthetic process	BP
PTTG_03037	Uncharacterized protein	GO:0035556	Intracellular signal transduction	BP
PTTG_03037	Uncharacterized protein	GO:0016849	Phosphorus-oxygen lyase activity	MF
PTTG_03037	Uncharacterized protein	GO:0004016	Adenylate cyclase activity	MF
PTTG_05519	Uncharacterized protein	GO:0006596	polyamine biosynthetic process	BP
PTTG_07059	Uncharacterized protein	GO:0035091	Phosphatidylinositol binding	MF

*BP, biological process; MF, molecular function*.

To further understand the potential impact of the NSY mutations on the candidates, we also inspected the predicted aa changes and assessed whether these NSY substitutions altered the aa characteristics. Eighty-four NSY mutations were identified for the 20 candidates, 70 of which led to a change in the aa characteristics (Additional File 5). Almost all the candidates genes except for 4 (PTTG_00930, PTTG_00931, PTTG_11639, and PTTG_06325) harbored at least one NSY mutation resulting in the characteristic change of aa. As such alterations were more likely to influence protein structure and function, they were highlighted in Additional File 5.

### The expression of *Lr20* candidates during *in planta* interaction

A gene expression analysis of pair 9 isolates 630550 (avirulent on *Lr20*) and BCL-75 (virulent on *Lr20*) inoculated on Chinese Spring (Cs without *Lr20*) and the near isogenic line Cs/Ax (with *Lr20*) was carried out as a pilot investigation of the gene expression of the 20 candidate effectors during *in planta* interaction. The transcriptome analysis showed that 17 candidate genes had detectable expression ranging from >100 RPKM (reads per kilobase per million mapped reads; 8 candidates) to < 5 RPKM (4 candidates). The fold change of the gene expression in BCL-75 (*n* = 2; 1 Cs, and 1 Cs/Ax) vs. 630550 (*n* = 2, 1 Cs and 1 Cs/Ax) is listed in Table 6. Six and three genes exhibited ≥1.2 fold down- and up-regulation in BCL-75 vs. 630550, respectively, and two genes (PTTG_25257 and PTTG_30778) had low expression level in only one isolate (Table 6). These results, while providing additional information to narrow down the potential *Lr20* effectors, should be interpreted with caution due to the small sample size of the isolates. Further transcriptome studies using larger sample size across the majority of the 20 isolates in a time course manner are thus warranted.

**Table 6:** **Expression of ***Lr20*** candidates in pair 9 isolates during ***in planta*** interaction**.

**Gene ID**	**Fold Change BCL75 vs. 630550**	**BCL75-Cs-RPKM**	**BCL-75-Cs/Ax-RPKM**	**BCL-75—Means**	**630550-Cs-RPKM**	**630550-Cs/Ax-RPKM**	**630550—Means**	**Amino acid size**
PTTG_00930	−1.2	143.57	114.9	129.24	128.89	177.23	153.06	196
PTTG_00931	−1.2	490.86	428.66	459.76	483.57	618.89	551.23	209
PTTG_01476	−1.1	8.88	9.55	9.22	10.53	9.32	9.93	521
PTTG_03715	−1.5	100.51	119.61	110.06	188.48	139.89	164.19	453
PTTG_03824	3.6	0.3	0.07	0.18	0.1	0	0.05	401
PTTG_03866	−1.2	167.87	181.25	174.56	210.26	207.5	208.88	499
PTTG_06324	1.1	532.53	451.83	492.18	392.15	489.76	440.95	296
PTTG_06325	−1.8	102.79	106.04	104.41	160.86	210.77	185.82	280
PTTG_06625	1.8	6.97	6.15	6.56	3.74	3.52	3.63	504
PTTG_08794	1.3	473.79	436.5	455.14	294.98	390.38	342.68	134
PTTG_09239	1.0	114.52	145.78	130.15	164.66	90.41	127.53	190
PTTG_11639	−2.1	1.62	2.52	2.07	4.25	4.62	4.43	226
PTTG_25257	NA	0	0	0	0.22	0	0.11	125
PTTG_25496	1.1	21.19	23.77	22.48	18.88	21.79	20.33	180
PTTG_26540	1.0	49.71	48.48	49.09	49.81	45.32	47.56	520
PTTG_29866	1.0	5.02	4.57	4.79	5.87	3.46	4.67	805
PTTG_30778	NA	0.19	0	0.09	0	0	0	212

*Cs, Chinese spring; Cs/Ax, Chinese spring near isogenic line*.

*BCL-75: pair 9, Lr20 virulent; 630550: pair 9, Lr20 avirulent*.

*Fold change: “−” and “+” indicated down- and up-regulation in BCL-75 vs. 630550*.

*RPKM, reads per kilobase per million mapped reads*.

*NA, not applicable*.

### Copy number and structural variants in relation to the expression of *Lr20* candidates

To examine whether the altered gene expression could be affected by copy number variation (CNV) or other structural rearrangements (e.g., inversion and translocation), an analysis of CNV and structural variants was carried out and the 20 candidate genes in pair 9 isolates were further inspected. No copy number or structural variant was detected for all of the candidate genes except for PTTG_06625 at 464,196–465,868 bp on supercontig 18, which was encompassed by an inversion. This 89,279 bp (387,749–477,028 bp) inversion covering six genes with PTTG_06625 and PTTG_26286 located in the middle relative to the other genes was present in both isolates 630550 and BCL-75, but absent in the remaining isolates. As a recent study on yeast has shown that small sized chromosomal inversions (< 100 kb) encompassing few genes are pervasive (Naseeb et al., 2016) and we have detected the 89 kb inversion in both avirulent and virulent isolates, it is unlikely that this event would be the main contributor of the up-regulation (1.8 fold) of PTTG_06625 in the virulent isolate (Table 6).

### Biological functions of the derived genes with GO annotation

As the genes identified from the aforementioned approaches, including SNP association analysis, inspection of more than 10 NSY SNPs, and differential counts in NSY SNPs by the Wilcoxon rank sum test, could be directly relevant in *Pt* pathogenicity, they were examined in greater detail. A wide range of biological processes were covered by these annotated genes, which were grouped broadly into five major categories: (1) carbohydrate metabolism (e.g., polysaccharide and cellulose catabolism); (2) macromolecular metabolism (e.g., DNA recombination, transcription regulation, ubiquitin-dependent protein degradation, and serine family metabolism); (3) cellular component organization and signal transduction (e.g., Golgi organization, microtubule cytoskeleton organization, Rho protein signal, TOR signaling, signal peptide processing, and cAMP biosynthesis); (4) energy generation, lipid metabolism and oxidation-reduction (e.g., ATP catabolism, NAD biosynthesis, and phosphatidylinositol); and (5) transport (e.g., cation, metal ion, protein, and phospholipid transport; Additional Files 2, 3; Figure 3).

## Discussion

To understand the molecular mechanisms of the *Pt*-wheat interactions related to pathogenicity for *Lr20*, we sequenced the genomes of 20 *Pt* isolates, comprising 10 phenotype matched pairs with contrasting pathogenicity for this resistance gene (Table 1; Figure 1). Our study is not only the first to investigate genomic variation in a panel of phenotype-paired isolates of a rust pathogen, but also the first to attempt genetic association analysis for the prediction of avirulence genes in *Pt*. An average of 67 million DNA reads was generated and more than 350,000 SNPs were identified for each of the 20 samples (Tables 2, 3), which allowed both the construction of a whole-genome SNP-based phylogenetic tree (Figure 2) and the prediction of candidate effectors associated with *Lr20* avirulence (Table 4).

The reads of each isolate were mapped directly against the *Pt* Race 1 reference genome for SNP detection, in a similar way to several previous studies (Zheng et al., 2013; Persoons et al., 2014). An average of 404,690 SNPs per isolate (3 SNPs/kb) was found and the proportion of heterozygous SNPs was around 87% (2.6 SNPs/kb) in a majority of the isolates, which highlighted not only a high level of SNP, but also a high heterozygosity rate across the *Pt* genomes. This rate of heterozygous positions matches that reported for the *Pt* race 1 genome (2.57 SNPs/kb; Cuomo et al., 2016). This feature has been consistently noted in the genomes of other rust fungi, such as *M. larici-populina, Pgt*, and *Pst* (Cantu et al., 2011; Zheng et al., 2013; Persoons et al., 2014; Upadhyaya et al., 2014a). The frequency of heterozygous SNPs in our *Pt* genomes appears lower than that in isolates of *Pgt* from Australia (~10 SNPs/kb; Upadhyaya et al., 2014a) and of *Pst* from the UK/US (~6 SNPs/kb; Cantu et al., 2013), but higher than the rate of the Chinese *Pst* isolate CY32 (~1.0 SNP/kb; Zheng et al., 2013). As the SNP rate in *Pst* CY32 is likely to be underestimated due to the use of fosmid clones and a resulting separate assembly of allelic regions from the two haplotypes, it appears likely that *Pt* may exhibit a lower frequency of heterozygous SNPs than both *Pgt* and *Pst*.

Interestingly, although the total number of SNPs varied from 329,300 to 446,048, the ratio of intergenic vs. total SNPs, and synonymous, and nonsynonymous SNPs vs. coding SNPs in each isolate remained almost the same (83, 37, and 61%, respectively; Table 3). This is consistent with the recent finding that these three types of mutation in an asexual population of yeast were fixed at around the same rate in about 1000 generations (McDonald et al., 2016). Given that *Pt* reproduces asexually in Australia (Park et al., 1995), the ratios we observed here in clonal lineages may actually reflect the similar ratios of the intergenic, synonymous, and non-synonymous mutations in presumed founding isolates. It has been further argued that unlike sexual populations, adaptation in asexual populations is limited by competition between cohorts with linked mutations from the same genetic background, which drives some beneficial mutations to extinction (McDonald et al., 2016). Coincidently, one *Pt* pathotype detected in 1990 and not included in the present study involves loss of virulence for *Lr20* (Park et al., 1999). This could be an example of beneficial mutations being swept out in asexual populations, and thus warrants further investigation.

The genome-wide SNP-based phylogenetic tree revealed two distinct clades with the large clade consisting of 15 isolates and the small one consisting of the remaining five (Figure 2), which was also consistent with the PCA results (Figure 1 in Additional File 1). The small clade was characterized by *Pt* isolates with the lower heterozygosity level, which may reflect a higher level of inbreeding on the alternate host in the event that gave rise to the ancestor of these isolates. One branch of the small clade comprised isolates 60-L-2 and 64-L-3 (pair 7), which showed greatest genetic distance to all other isolates, the lowest number of total SNPs (both < 350,000), and the lowest frequency of heterozygous SNPs (Table 3). The other branch consisted of the isolates 900273, 900084 (pair 13), and 89-L-1 (pair 14), which harbored the largest number of total SNPs (all >435,000; Table 3) with the second lowest heterozygosity level (all ~81%; Table 3). The lowest ratios of heterozygous SNPs in pair 7 isolates were likely to be an intrinsic feature of this clonal lineage as the coverage of reads for one of the paired samples, 64-L-3, was the highest of all isolates.

In contrast, all isolates in the large clade showed a consistently higher percentage of heterozygous SNPs (~87%; Table 3), implying that they may have arisen from more diverse populations. In addition, it was noted that 670028 (pair 2; *Lr20* virulent) was genetically more distant from all of the other isolates in this clade, which may partially relate to it being collected in New Zealand (Table 1). It appeared that for each pair of isolates, those that formed sister groups in the phylogenetic tree were all from the same region, but not all the pairs from the same region clustered together (Table 1; Figure 2). This observation may imply that at the DNA level, isolates from different locations were more likely to be divergent, and that isolates from the same region with similar pathogenicity phenotypes were not necessarily closely related. Moreover, the SNP derived evolutionary relationships were not always related to the virulence features of the samples (Figures 1, 2), which may indicate that isolates with similar virulence phenotypes may have evolved from genetically different clonal lineages, representative of a transitory adaptive evolution status.

A set of 20 candidate effectors for *Lr20* was identified through an integrated approach that combined both genetic association with the *Lr20* virulence phenotype and structural features of fungal effectors (Table 4). To the best of our knowledge, for the analysis of whole genome sequencing, our study is the first to suggest such a combined approach, which has the potential to dramatically accelerate the process of effector identification in rust pathogens. Throughout our analysis, we took both alleles into account by including homozygous and heterozygous genotypes for all isolates (Table 1 in Additional File 1). As aforementioned, we detected phenotype-associated alleles shared in homozygous as well as heterozygous status in the 20 candidate effectors. The homozygous state is in agreement with most observations that virulence is usually recessive and the virulence gene is homozygous at the mutation point, which may result from the same mutation in both alleles or a deletion of one allele (Isaac, 1991; Anderson et al., 2016). On the other hand, the heterozygous state may indicate a dominant mutation as suggested by a previous study on two *Pt* populations in Canada (Kolmer, 1992). This study found that while all individuals from an uredinial population (asexual) were virulent to *Lr3* and *Lrl4a*, avirulent isolates were recovered in the aecial generation (sexual), indicating that virulence at these loci may be dominant. An alternative mechanism could be that a dominant inhibitor of avirulence at a second locus may account for the dominance of virulence (Haggag et al., 1973). Future studies at the protein level will be needed to clarify the biological functions in relation to zygosity status of the candidate effectors identified in this study.

As a pilot study, the validity of our method has been initially demonstrated by the small number of candidates we identified and the convergence of some predicted effectors from this study with those from previous studies on transcriptome and proteome of *Pt* (Song et al., 2011; Bruce et al., 2014; Rampitsch et al., 2015). Coincidently, a recent study has also argued that association analysis can be a powerful tool for the identification of AVR genes (Xia et al., 2016). By using 97 secreted protein-single nucleotide polymorphism (SP-SNP) markers in a natural *Pst* population of 352 isolates with various virulence phenotypes, this study successfully identified 30 SP-SNPs associated with nine *Pst* virulence phenotypes. However, this study was limited by a relatively small number of SNP markers and some unbalanced distributions of avirulence and virulence phenotypes, whereas ours was constrained by the relatively small sample size. Thus, to fully realize the potential of the association tool in avirulence effector identification, a larger number of isolates with balanced phenotypes should be selected and genotyped through whole genome sequencing. Such an approach should greatly accelerate parallel studies on genetic variation, evolution, and identification of avirulence genes.

Consistent with previous reports, most of the candidate avirulence genes identified in the present study encode hypothetical proteins with unknown functions (Table 4). Only two genes, PTTG_01476 and PTTG_03866, were predicted to be involved in proteolysis and carbohydrate metabolism, respectively. Meanwhile, at the gene expression level, our transcriptome study on the pair 9 isolates has shown that 17 of these candidates had detectable expression and both up- and down- regulation were observed in the candidates in the isolate BCL-75 (virulent on *Lr20*) vs. the isolate 630550 (avirulent on *Lr20*). While the down-regulation of the candidates may be related to the evasion of the host recognition by *Lr20*, the up-regulation of the candidates may be related to the introduction of new effectors or regulators resulting in virulence. However, limited by the small sample size, these results only provided a clue to the likely changes of the gene expression; a more statistically robust quantitative expression analysis will be carried out in future, which will use the majority of the isolates for mRNA sequencing over a time course.

In addition to these candidates, the whole list of the derived genes (SNP association, Wilcoxon rank sum test, and NSY SNPs >10; Table 5; Additional Files 2–4) that could be annotated revealed a wide range of biological processes highly relevant to rust pathogenicity and the biotrophic life history, as implicated in previous studies (Figure 3; De Wit et al., 2009; Lo Presti et al., 2015). Furthermore, the five major functional categories reflected by the annotated genes were consistent with previous studies on rust fungal genomes, showing that specific gene families including helicases, peptide transporters and different types of glycosyl hydrolases, lipases, and peptidases, were particularly expanded (Duplessis et al., 2011; Huang et al., 2011; Zheng et al., 2013). It has also been noted that the aforementioned biological themes may cover characteristic processes spanning all life stages of rust fungi, including the up-regulated processes highlighted in germinating spores such as cell proliferation, DNA, and cell wall metabolism and those representative of haustorial stage, such as energy production and biosynthetic processes (Garnica et al., 2013).

Our data also suggest the potential involvement of epigenetics and small RNA in *Pt* pathogenicity. One of the annotated genes out of the whole list of derived genes, PTTG_ 01525, was found to contain a domain (MT-A70) of methyl-transferase (Table 4 in Additional File 3), an enzyme that sequence-specifically methylates adenines in pre-mRNAs. Although the functional significance of this modification remains unknown, a similar mRNA methyl-transferase in yeast (IME4) was shown to be crucial for induction of sporulation (Clancy et al., 2002). In addition to RNA methylation, a recent study on *Magnaporthe oryzae* suggests that DNA methylation in fungi can also be a dynamic epigenetic entity contributing to fungal development and genome defense (Jeon et al., 2015). At the post-transcription level, the detection of 3′ UTR SNPs may implicate a role of small RNA-mediated gene expression in rust pathogenicity, well in line with the previous research on *Pst* that predicted endogenous small *Pst*-RNAs may target fungal and/or wheat genes for post-transcriptional silencing involved in stripe rust pathogenicity (Mueth et al., 2015).

A few limitations of this study should be noted. Firstly, Race 1 on which the reference genome is based is virulent on *Lr20* (through personal communication), and the effector prediction of the present study is based on the assumption that the avirulence gene for *Lr20* is not completely deleted in Race 1. In future studies, an isolate containing the *Lr20*- avirulence gene will be sequenced using long-read sequencing technologies to build a new reference genome assembly of better quality. Secondly, while we chose to focus on SNP variations in this study which reflects an important layer of genetic variations contributing to the virulence, CNV could be another important factor worth exploring. In future, long-read sequencing technology will be able to generate more reliable CNV calls as more precise mapping of long reads can reduce mapping errors, increase the sensitivity of CNV detection in repetitive regions, and ameliorate the inherent limitations of short-read sequencing (Zhao et al., 2013). Thirdly, this study used a relatively small sample size appropriate for the pilot investigation. Future studies using a larger sample size are thus warranted to further confirm and refine the candidate genes associated with avirulence gene to *Lr20*. Finally, the association analysis in this study was preliminary, and did not take kinship or group stratification into consideration due to the small sample size. Nevertheless, our results have shown that association analysis can be a powerful filter for avirulence gene identification. Future studies in a homogenous population with more samples shall be able to fully realize the potential of association analysis of rust pathogens.

In summary, our whole genome sequencing of 20 *Pt* isolates comprising pairs that contrasted in pathogenicity for *Lr20* not only revealed high genetic variation and heterozygosity rate across the genomes, but also predicted a small number of candidate effectors related to *Lr20*. For the first time, at the genome wide scale, genetic association with virulence phenotype integrated with protein structure prediction has been demonstrated as a promising tool for the identification of candidate effectors in rust fungi. Functional studies on these candidates are warranted to investigate their potential role in pathogenicity. One plausible approach is to deliver the candidate effectors into wheat leaf cells, based on the type III secretion system using *P. fluorescens* effector-to-host analyser strain, in a similar way to the previous study of screening the *Pgt* candidate effectors (Upadhyaya et al., 2014b). Moreover, integrated genomics approach of transcriptome, methylom, and small RNA profiling in future will further unveil the immune networks underlying *Pt-*wheat interactions, which will ultimately lead to more effective surveillance and management strategies for one of the most devastating pathogens of wheat.

## Materials and methods

### *Puccinia triticina* isolates

Twenty Australian isolates representing 20 pathotypes within seven *Pt* races (10, 26, 122, 135, 162, 104, and 68) were used in this study (Table 1). These isolates formed 10 pairs, with each pair comprising isolates differing only in avirulence/virulence to *Lr20*. All isolates were collected between 1974 and 1991 from the field, and the demographic map showing location and region of the collection sites was constructed using software ArcGIS (Esri, USA). The samples were isolated and maintained as viable cultures in liquid nitrogen at Plant Breeding Institute, Cobbitty, NSW, Australia (Park, 1996). To ensure isolate purity, a single pustule from a low density infection was subcultured from each isolate and propagated on the wheat cultivar Morocco in isolation prior to DNA preparation. For RNA isolation, 630550 (pathotype 135-2) and BCL-75 (pathotype 135-1, 2) were propagated on both wheat cultivars, Chinese Spring (CS without *Lr20*) and the near isogenic line Cs/Ax (with *Lr20*). The identity and purity of each isolate were checked by pathogenicity tests with a set of host differentials. For rust infection, host plants were grown at high density (~25 seeds per 12 cm pot with compost as growth media) to the two leaf stage (~7 days) in a growth cabinet set at 18–25°C temperature and 16 h light. Spores (−80°C stock) were first thawed and heated to 42°C for 3 min, mixed with talcum powder and dusted over the plants. Pots were placed in a moist chamber for 24 h and then transferred back to the growth cabinet. For DNA isolation, mature spores were collected, dried and stored at −80°C.

### DNA isolation from *Pt* urediniospores and genome sequencing

DNA was extracted from urediniospores by a CTAB extraction method (Rogers et al., 1989) with some modifications, including the use of 0.5 mm glass beads instead of fine sand and dry beating (2 × 1 min) at full speed on a dental amalgamator instead of grinding in liquid nitrogen. Extraction was carried out in several batches each with ~50 mg of dry spores and equal volume of 0.5 mm glass beads to accumulate sufficient quantities of DNA from different isolates. After CTAB extraction, samples were treated with DNase-free RNAase, extracted with phenol/chloroform/isoamyl alcohol (25:24:1) and purified using Qiagen Genomic tips (cat No 10262, Qiagen). DNA quality was assessed using the Bioanalyzer 2100 (Agilent Technologies). DNA from urediniospores of 20 *Pt* isolates was sequenced using the Illumina HiSeq2000 platform (101 bp paired-end reads) at the Broad Institute. Genomic DNA was sheared using a Covaris LE instrument and 180 base fragments adapted for sequencing as previously described (Fisher et al., 2011). Raw sequence reads generated and used in this study will be available in NCBI under BioProject PRJNA343337.

### RNA isolation and sequencing

Infected leaves were collected 7 days after inoculation and immediately frozen in liquid nitrogen. Samples were ground to a fine powder in liquid nitrogen and total RNA was isolated with the RNeasy Plant Mini Kit (Qiagen). After DNase treatment (Promega), RNA was further purified using the RNeasy Plant Mini Kit columns and the quality was assessed using the Bioanalyzer 2100. For library preparation, around 10 μg of total RNA was processed with the mRNA-Seq Sample Preparation kit (Illumina). Libraries were prepared for the pair 9 isolates, including BCL-75 (1 for Cs and 1 for Cs/Ax) and 630550 (1 for Cs and 1 for Cs/Ax). Each library was sequenced using the Illumina HiSeq2500 platform (125 bp paired-end reads).

### Genome alignment and SNP calling

Paired Illumina reads of the 20 isolates were independently aligned against the reference assembly for *Pt* Race 1 using BWA v0.5.9 with default options (Li and Durbin, 2009). High quality alignments (with the mapping quality of at least 30) were selected using the SAMTools view command. These BAM files were sorted and indexed and used for SNP calling with GATK v2.1.9. To minimize false positives around insertion/deletions (indels), regions around indels were identified using the GATK RealignerTargetCreator. With the indel intervals defined, the GATK IndelRealigner was implemented on the high quality BAM alignment files. The re-aligned BAM generated was then used as input for GATK UnifiedGenotyper to call variants between the reference assembly and sequence reads. To remove low confidence calls, the unfiltered SNP data were filtered using GATK VariantFiltration module, with threshold values as the following: Fisher Strand >60.0, Haplotype Score > μ+2σ, Mapping Quality < 30.0, Mapping Quality Rank Sum < −12.5, Quality by Depth < 2.0, and Read Position Rank Sum < −8.0. These were recommended as the “best practices” at the time the SNP calling was carried out. As the *Pt* genome is highly repetitive, a maximum coverage threshold to limit false SNP discovery due to paralogous SNPs was also applied (positions with coverage > 1.5 times the median coverage of the entire genome removed). The identified SNPs were then annotated using VCFannotator (http://vcfannotator.sourceforge.net/) based on the *P. triticina* gene set (Cuomo et al., 2016). To evaluate whether the NSY mutation in the candidates resulted in any change in aa characteristics, aa residues were classified as the following 6 characteristic groups: (1) aa with hydrophobic side chain—aliphatic (A, I, L, and V); (2) aa with hydrophobic side chain—aromatic (F, W, and Y); (3) aa with polar neutral side chains (N, C, Q, M, S, and T); (4) aa with electrically charged side chains—acidic (D and E); (5) aa with electrically charged side chains—basic (R, H, and K); and (6) unique aa (G and P). NSY mutations resulted in the change of aa characteristics were further highlighted in Additional File 5.

### Transcriptome and structural variants analyses

For transcriptome analysis, quality trimmed (0.01 quality trim, minimum length 50) RNA reads from isolates 630550 and BCL-75 were first aligned against the Race 1 reference gene set using the CLC Genomics Workbench module RNA-Seq Analysis (default parameters). Expression levels were then quantified as RPKM for comparison between BCL-75 and 630550. For the CNV and structural variant analyses, the module of Structural Variants Tool in CLC Genomics Workbench (default parameters) was used.

### Phylogenetic relationship

Phylogenetic relationships based on heterozygous and homozygous SNP differences were inferred using PAUP^*^ Maximum Parsimony and “hetequal” substitution model (Swofford, 2002). In order to build a tree based on high quality data, only those SNP sites that had no more than 5% missing information across the 20 isolates were included. A total of 578,967 SNP sites were extracted and a tree with 270,784 parsimony-informative characters and 1000 bootstrap replicates was constructed, as shown in Figure 2B. The PCA analysis was performed using smartpca from eignesoft (https://github.com/DReichLab/EIG).

### Statistical analyses and biological annotation

SNP association analysis was carried out using the sample label swapping with a max(T) permutation approach implemented in plink (Purcell et al., 2007; Chang et al., 2015). Wilcoxon rank sum test was employed to identify genes with the total number of non-synonymous changes significantly different between the *Lr20* avirulent and virulent groups. As UTR length in *Pt* has not been characterized in detail, the 1000 bases upstream and downstream of the CDS were selected as UTRs, which may contain some intergenic sequences. Heatmap.2 from gplots package, one of the R programming tools for plotting data, was used to generate heatmaps. Genes either harboring at least one SNP with *p* < 0.05 or showing differential NSY SNP counts between groups were then subjected to structure predictions of signal peptide (D score cutoff value: 0.45) and transmembrane segment using SignalP v4.0 with the SignalP-TM network function (Petersen et al., 2011) and TMHMM (Krogh et al., 2001), respectively. Those genes encoding proteins with a signal peptide and no transmembrane segment were identified as effector candidates. For GO annotation, genes of interest were submitted to the Fungifun (Priebe et al., 2011, 2015) and the Venn diagram was constructed using Venny v2.1 (BioinfoGP).

## Author contributions

JW analyzed the data and wrote the manuscript; SS analyzed the data and contributed to the manuscript; CD and PZ did the RNA isolation; RP and CC supervised the work and revised the manuscript; RP designed the experiment. All authors read and approved the final manuscript.

### Conflict of interest statement

The authors declare that the research was conducted in the absence of any commercial or financial relationships that could be construed as a potential conflict of interest.
